# Tolerance and surface analysis of veterinary bone screws

**DOI:** 10.3389/fvets.2026.1723402

**Published:** 2026-02-11

**Authors:** William T. McCartney, Ciprian Ober, Bryan J. Mac Donald, Christos Yiapanis

**Affiliations:** 1 NOAH, Dublin, Ireland; 2 Department of Surgery and Intensive Care, Faculty of Veterinary Medicine, University of Agricultural Sciences and Veterinary Medicine, Cluj-Napoca, Romania; 3 School of Mechanical and Manufacturing Engineering, Dublin City University, Dublin, Ireland; 4 School of Veterinary Medicine, University of Nicosia, Egkomi, Cyprus

**Keywords:** analysis, bone, implant, screw, surface, tolerance, veterinary

## Abstract

**Introduction:**

While dimensional accuracy and surface characteristics of human orthopedic implants are extensively regulated and studied, comparable evaluations of veterinary orthopedic implants are limited. This study aimed to assess the dimensional conformity and surface finish of commercially available veterinary cortical bone screws relative to established engineering standards.

**Methods:**

Seventy-three commercially available 2.0 mm veterinary cortical bone screws from five anonymized manufacturers were analyzed using high-magnification optical microscopy. Dimensional parameters assessed included screw length, major diameter, minor diameter, thread pitch, and combined thread angle (α + β). Measurements were calibrated using a certified micrometer scale. Dimensional conformity was evaluated using a unilateral tolerance framework based on ISO 5835 low-quality limits. Surface finish was assessed using a semi-quantitative grading system under standardized magnification.

**Results:**

Most screws exhibited dimensional deviations outside the defined tolerance limits. Eighty percent of screws were outside tolerance for length, 78% for major diameter, 77% for minor diameter, 98.6% for pitch, and 60% for combined thread angle. Surface finish was classified as unacceptable in 40% of screws. Tolerance deviations were observed across all manufacturers, with no single manufacturer accounting for the majority of non-conforming screws.

**Discussion:**

The findings demonstrate substantial dimensional variability and surface defects in commercially available veterinary cortical bone screws. These deviations may adversely affect screw biomechanics and the bone–implant interface, indicating suboptimal manufacturing quality. The absence of regulatory oversight for veterinary implants highlights the need for improved quality control standards to ensure consistent and reliable implant performance.

## 1 Introduction

Human orthopedic implants undergo extensive analysis, not only for research purposes but also to satisfy regulatory requirements, such as CE marking. Standardized testing protocols are outlined in ASTM and ISO documentation (1–7). In addition to regulatory testing, extensive research studies have been conducted on human orthopedic implants (8–13), focusing on the geometric and mechanical parameters of bone screws, including pitch, length, angle, and material (10–12). All aspects of bone screw design and usage have been investigated, with particular attention to the influence of the pilot hole on screw holding power (14–16). The design of self-tapping cutting flutes significantly affects insertion torque (17), and incorrect torque can compromise fixation strength (18–20). Metal degradation in tissues has also been reported, with retrieved screws showing weight loss after implantation (21).

Optimal screw geometry depends on bone density, as pitch and other parameters vary accordingly (22). Despite correct insertion, bone screws often do not achieve uniform contact with surrounding bone, with thread-to-bone gaps reaching up to 130 μm and intra-screw thread diameter variation ranging from 70 to 180 μm (23). Studies using metrology have shown that some screws fall outside recommended dimensional tolerances (24), and similar inconsistencies have been observed in dental implants (25). Manufacturing of orthopedic screws is complex, involving multiple machining steps such as turning, shaping, drilling, milling, sawing, grinding, and broaching, which can introduce deviations (26–29). Additive manufacturing is currently impractical for mass production due to speed and consistency limitations, although future robotic-assisted systems may overcome these challenges (29).

In contrast, veterinary orthopedic implants are not subject to regulatory requirements, and no formal analysis is required before market release or after distribution. This regulatory gap is reflected in limited research on veterinary implant quality. Elemental analysis of veterinary stainless steel implants revealed that 41.6% of the tested implants were not the correct grade (316 vs. 316 L) (30), highlighting potential risks to patients despite reduced manufacturing costs.

## 2 Materials and methods

### 2.1 Specimens

A total of 73 commercially available 2.0 mm cortical bone screws were randomly selected from routine clinical stock. Screws originated from five different manufacturers, which were anonymized for analysis purposes (M1–M5). Manufacturer identity was recorded at the time of analysis, but batch or lot numbers were not consistently available and were therefore not included. No ethical approval was required for this study, as it involved only the analysis of commercially available, unused implants and did not include live animals or human subjects.

### 2.2 Microscopy and calibration

**Figure 1:**
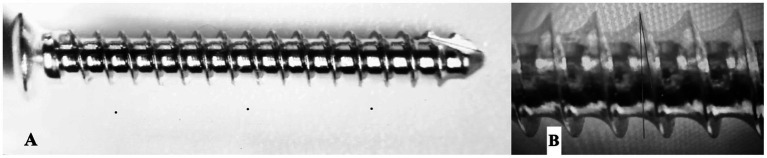
Measurement landmarks and geometric definition of thread parameters. **(A)** Full-length view of a 2.0 mm cortical screw illustrating the mid-shaft region used for dimensional measurements, avoiding the head–shaft transition and distal tip. **(B)** Optical microscopy image of the screw thread profile illustrating the geometric construction used for measurements. Pitch was measured as the crest-to-crest distance along the screw axis and averaged over three consecutive threads. Thread flank angles (α and β) were defined on a two-dimensional projection, and the combined thread angle (α + β) was used for analysis.

Measurements were performed using an Insize ISM-PM200SA digital microscope equipped with ISM-PRO 2.00.0002.01 software. Dimensional measurements were performed using high-magnification optical microscopy (Leica DMS1000, Leica Microsystems, Germany) at magnifications of 50× and 100×. Images were acquired at a native resolution of 1,920 × 1,080 pixels. Prior to measurement, the system was calibrated using a certified stage micrometer with 10-μm graduations, yielding a measurement precision of ±2 μm. Measurement locations and geometric construction of pitch and combined thread angle are illustrated in Figure 1. All measurements were performed using the manufacturer’s image analysis software by a single operator under identical imaging conditions.

Major diameter (D1) was measured as the crest-to-crest distance of opposing threads at the mid-shaft region of the screw, avoiding the head–shaft transition and the distal tip. Minor (core) diameter was measured as the root-to-root distance between opposing thread valleys at the same mid-shaft region. Thread pitch was defined as the axial distance between consecutive thread crests measured parallel to the screw axis. Pitch was calculated as the mean of three consecutive crest-to-crest distances measured at the mid-shaft region of each screw. Thread angle was assessed on a two-dimensional projection by constructing straight lines along the leading (α) and trailing (β) flanks of a representative thread. The combined thread angle (α + β) was calculated as the sum of these two angles, consistent with ISO 5835 methodology.

### 2.3 Screw positioning and dimensional measurements

Each screw was positioned on the microscope platform with the shaft aligned parallel to the stage to minimize measurement errors due to tilting. The nominal major diameter (D1) of the evaluated screws was 2.00 mm. Because no veterinary-specific dimensional tolerances are defined for cortical bone screws, a unilateral (negative-only) tolerance framework was adopted, using the ISO 5835 “low-quality band” as a conservative reference. Under this framework, measurements equal to or below the nominal value but not exceeding a −5% deviation were considered within tolerance, while any positive deviation (oversizing) was classified as out of tolerance. This standard was applied consistently to all other measured dimensions. The combined thread angle (α + β) was defined as the combined alpha and beta angles, set at 38°.

### 2.4 Tolerance framework and pass/fail rules

For all dimensional parameters, a nominal target value was defined based on manufacturer specifications for 2.0 mm cortical screws. Because no formal dimensional tolerances exist for veterinary implants, a unilateral tolerance model was applied using the ISO 5835 low-quality tolerance band as a conservative reference. Under this approach, only negative deviations (undersizing) up to 5% below the nominal value were considered acceptable. Any measurement exceeding the nominal target value (positive deviation) was classified as out of tolerance. The operational pass/fail limits applied in this study were as follows: screw length: target 14.00 mm; acceptable range 13.30–14.00 mm, major (outer) diameter, D1: target 2.00 mm; acceptable range 1.90–2.00 mm, minor (core) diameter: target 1.40 mm; acceptable range 1.33–1.40 mm, thread pitch: target 0.50 mm; acceptable range 0.475–0.50 mm, combined thread angle (α + β): target 38.0°; acceptable range 36.1–38.0°. Measurements falling outside these ranges were classified as out of tolerance. Surface characteristics were evaluated qualitatively; screws showing major surface defects (e.g., deep machining grooves, burrs, or metal flaking) were classified as unacceptable regardless of dimensional conformity. For thread pitch, conformity was evaluated relative to both the ISO 5835 reference value (0.5 mm) and the commonly reported veterinary literature value (0.6 mm), allowing comparison of outcomes across reference standards.

Surface integrity was assessed using a semi-quantitative ordinal grading system ranging from 1 to 3, where higher grades indicate greater defect severity (Table 1). All surface evaluations were performed by a single trained observer using calibrated high-magnification optical microscopy under consistent imaging conditions. The observer was not blinded to screw type. No inter-rater agreement analysis was performed. When multiple defect types were present on a single screw, the final surface grade corresponded to the highest observed defect severity.

**Table 1:** Semi-quantitative rubric for surface defect grading.

Grade	Machining marks	Burrs/edge defects	Surface debris/flaking	Acceptability
1 (Minor)	Fine, uniform machining lines without interruption	Absent or minimal, not protruding	None visible	Acceptable
2 (Moderate)	Pronounced machining marks with local irregularity	Small burrs present, not sharp	Localized debris or coating irregularity	Acceptable with caution
3 (Major)	Deep or irregular machining grooves	Sharp or protruding burrs	Metal flaking, delamination, or loose debris	Unacceptable

### 2.5 Operator validation

Measurement accuracy was validated by repeated measurements at identical locations, yielding mean variances of +0.02 mm for 0.1 mm measurements, +0.03 mm for 2 mm screw major diameter, and +0.01 mm for 2 mm screw pitch.

### 2.6 Surface finish assessment

**Figure 2:**
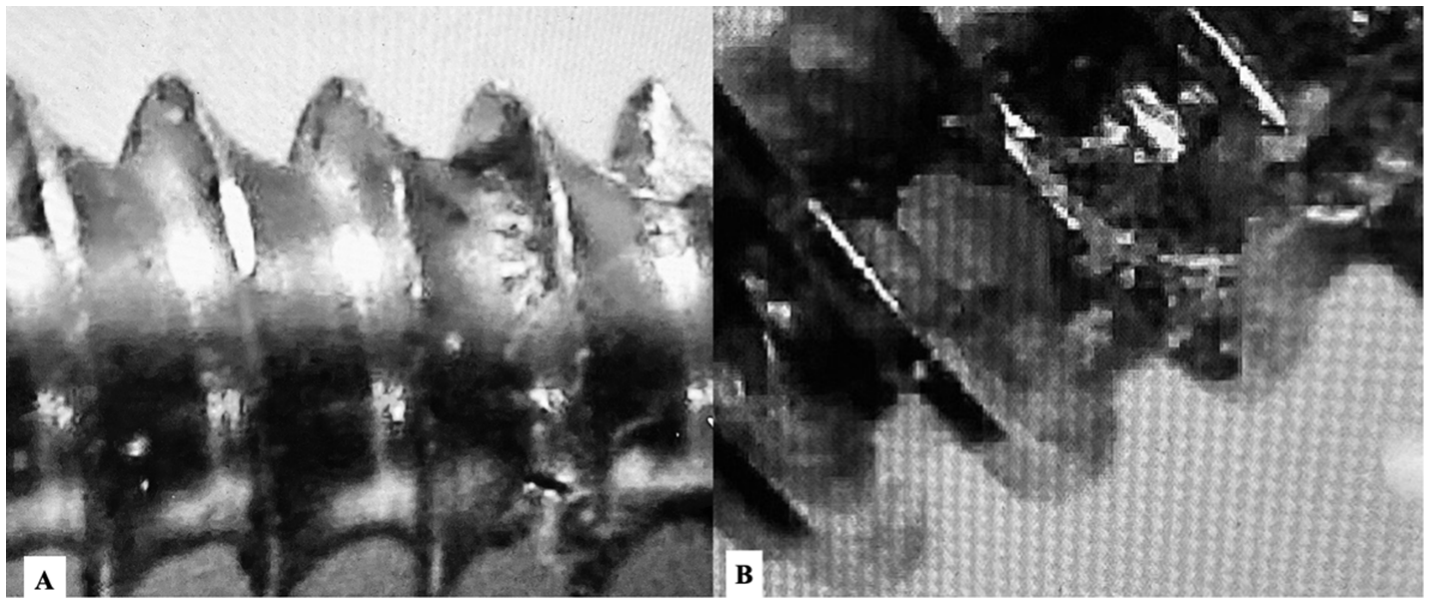
Representative optical micrographs illustrating surface finish grading. **(A)** Example of acceptable surface finish (Grade 1–2), showing uniform machining marks without major burrs, flaking, or thread deformation. **(B)** Example of unacceptable surface finish (Grade 3), characterized by pronounced surface irregularities, disrupted thread crests, and material defects.

Surface finish was evaluated visually under magnification for the presence of marks, scratches, dents, debris, pits, or direct damage. Each parameter was graded on a scale of 1 to 3, with 1 indicating minor defects and 3 indicating major defects. The degree of metal polish was also graded using the same scale. A human cortical screw was used as a reference standard to facilitate consistent grading of surface finish. Representative examples of acceptable and unacceptable surface finish grades are shown in Figure 2.

### 2.7 Statistical analysis

Confidence intervals (95%) were calculated for proportions only, corresponding to the percentage of screws within or outside tolerance limits. Proportion confidence intervals were computed using the Wilson score method, which provides improved accuracy for small sample sizes and extreme proportions. Continuous dimensional variables were summarized using descriptive statistics but were not associated with confidence intervals.

## 3 Results

Individual screw-level raw dimensional measurements are provided in Supplementary Table S2A, while tolerance conformity (Y/N) and surface acceptability classifications are reported separately in Supplementary Table S2B, following restructuring of the original dataset to separate measurements from interpretation.

### 3.1 Screw length

The nominal screw length was 14 mm. Only 35% of screws were within the tolerance range, and only one screw measured exactly 14 mm.

### 3.2 Major (nominal) diameter

The nominal diameter was 2 mm. Only one screw met this target, with 51 screws (70%) falling outside the tolerance range.

### 3.3 Minor diameter

Four screws were on target, and 22 screws were within tolerance. Overall, 68.5% of screws were outside the tolerance range.

### 3.4 Pitch

No screws measured exactly on target, and only 1 screw (1.4%) was within tolerance. When pitch conformity was assessed using a veterinary literature reference value of 0.6 mm instead of the ISO-based 0.5 mm target, a substantially higher proportion of screws fell within tolerance. This marked difference highlights the sensitivity of pitch conformity outcomes to the selected reference standard.

### 3.5 Combined thread angle (α + β)

No screws met the exact target angle, while 10 screws (13.7%) were within tolerance.

### 3.6 Surface finish

Surface analysis revealed that 29 screws (39.7%) had visible marks, with 8 screws (10.9%) exhibiting significant surface defects. Compared to a human cortical screw reference, the veterinary screws did not achieve the same level of polish.

Table 2 summarizes the number and percentage of screws meeting each objective, along with corresponding confidence intervals.

**Table 2:** Confidence intervals (95%) for proportions were calculated using the Wilson score method.

Outcome	*n*/*N*	% (95% CI)
Length with limits	26/73	36% (25, 48%)
Major diameter within tolerance limits	22/73	30% (20, 42%)
Core diameter within tolerance limits	23/73	32% (21, 43%)
Pitch within tolerance limits	1/73	1% (0, 7%)
Combined thread angle (α + β) within tolerance limits	10/73	14% (7, 24%)
Major damage to surface finish	8/73	11% (5, 20%)
Any damage to surface finish	29/73	40% (28, 52%)

The results indicate that for the first five parameters, relating to measurements within the tolerance limits, fewer than half of the screws met the target for each parameter. Approximately one-third of screws were within the desired limits for length, major diameter, and core diameter, whereas only 1% of screws were within the tolerance for pitch. Surface analysis showed that 40% of screws exhibited some damage, with 11% displaying major defects.

Tolerance deviations were observed across all anonymized manufacturers, with no single manufacturer accounting for the majority of out-of-tolerance measurements (Table 3).

**Table 3:** Manufacturer-level distribution of screws and tolerance outcomes.

Manufacturer	N screws	Median major diameter (mm)	% out of tolerance (any parameter)
M1	15	2.05	86.7
M2	14	2.01	78.6
M3	16	2.08	93.8
M4	14	1.98	71.4
M5	14	2.03	85.7

Descriptive statistics for all measured dimensional parameters are summarized in Table 4. Continuous variables are reported as mean ± standard deviation and minimum–maximum values to illustrate the distribution and variability of measurements.

**Table 4:** Descriptive statistics of measured screw dimensions (*n* = 73).

Parameter	Mean ± SD	Min–max
Length (mm)	14.12 ± 0.31	13.30–14.62
Major diameter D1 (mm)	2.04 ± 0.07	1.87–2.18
Minor diameter (mm)	1.41 ± 0.05	1.32–1.52
Pitch (mm)	0.58 ± 0.04	0.50–0.66
Combined thread angle (°)	39.6 ± 2.1	35.8–44.2

## 4 Discussion

This study provides a quantitative quality-control analysis of veterinary cortical bone screws and places the findings in the context of established engineering and regulatory standards.

The concept of tolerance is a specific engineering principle. Engineers recognize that there will be variance in a product due to the small differences that occur at each step of a manufacturing process. These differences are unavoidable, but they must be defined to ensure the product is finished to an appropriate quality. When mass production is used, the challenge of maintaining the correct tolerance becomes significant. This effectively means that very minor differences will be accepted as long as they fall within a range deemed not to adversely affect the performance of the product. In the aerospace and human medical engineering sectors, the tolerance range is deliberately set very tightly, given the level of performance expected and the consequences of product failure. Achieving very tight tolerances increases the cost of manufacture and places intense quality demands on the production processes. Every step of manufacture must be finely controlled and monitored.

For human medical devices, tolerances are specifically mentioned in regulations, e.g., ISO 5835 for screws (6). This specifies that the major (nominal) diameter or D1 of a 2 mm bone screw should be 2.00 mm (top quality) but provides a tolerance range, meaning it is acceptable if the diameter of D1 is between 1.9 and 2.00 mm (considered low quality), while it is recommended that the diameter D1 “should” be between 1.95 and 2.00 mm (middle quality). For the purposes of this study, the authors chose 1.9 mm as the lowest dimension of the tolerance range, making the range 1.9–2 mm. Under ISO 5835 (6), allowable tolerance for all other dimensions is −0.05 mm. For this study, the chosen tolerance was set at 95% using the initial figure for the D1 parameter. As there are no regulations for veterinary implants, no designated tolerance exists. Choosing 95%, based on human screw implant tolerances, was considered reasonable. This brings the tolerance for the 2 mm screws in line with a low-quality human implant.

Previous veterinary literature commonly reports a thread pitch of approximately 0.6 mm for 2.0 mm cortical screws, whereas ISO 5835 specifies a nominal pitch of 0.5 mm for human cortical screws of the same diameter (31). Similar manufacturing-related dimensional variability has been reported in metrology studies of orthopedic and dental implants, supporting the broader relevance of the present findings beyond a single implant category. Because no harmonized veterinary standard exists, both reference values were considered relevant comparators for interpreting pitch conformity in the present study. There is no reason to accept a pitch of 0.6 mm, and the authors are unaware how this figure was derived. This may reflect either different interpretation or manufacturer variance. The dimension criteria are specified for human screws, but not for veterinary ones; therefore, the expected standard to aim for is assumed to be the human standard. Variance between manufacturers was not studied in this project, but it is evident that, when comparing manufacturers for the same screw, differences exist in the number of screws falling within the tolerance range. For the orthopedic surgeon purchasing screws, navigating manufacturer brochures is challenging, as all manufacturers claim the same level of quality control. The absence of oversight or stipulated dimensions creates a void in the market that can be exploited commercially. Pitch showed the lowest conformity rate in this dataset. This may reflect manufacturing variability between producers and/or the existence of a different nominal pitch used in parts of the veterinary market (commonly reported as 0.6 mm), which would systematically shift measurements outside a 0.5 mm tolerance framework. Recent metrological analyses of orthopedic and dental implants have similarly highlighted clinically relevant dimensional variability in commercially available screws, indicating that manufacturing tolerances remain an active concern in contemporary implant production (24, 25).

It is possible to underestimate the complexities of manufacturing bone screws. Lack of knowledge of the required stages can lead to misconceptions. Surface integrity is clinically relevant not only from a manufacturing standpoint, but also because machining grooves, burrs, and surface debris have been shown in recent studies to influence implant–tissue interaction, corrosion behavior, and overall implant performance (25, 29). There is a need for clear direction and specification of best practice, which is covered under ASTM and ISO regulations (6–11). In this study, surface finish was inadequate in over one-third of screws, and the authors chose to maintain a low grading threshold to allow for variation. Had the grading standards been stricter, many more screws would have failed. Consideration must be given to the fact that finishing an implant is not straightforward. Once the basic structure has been created by turning the stainless-steel foundation piece, the product must be thoroughly cleaned, which involves intense degreasing as well as general cleaning. Ultrasound cleaners and other methods are used to clean the implant. This step is crucial, as ineffective cleaning can lead to surface imperfections during the subsequent passivation phase. Any impurities present prior to passivation can cause surface damage. Specialized machinery is recommended for quality passivation, which is usually carried out by electropolishing. Screws have many crevices and recesses, making electropolishing the recommended method, although simple acid immersion can also be used as a less effective alternative. An example electropolishing procedure involves immersing screws in a bath of highly concentrated acids (96% sulfuric acid and 85% orthophosphoric acid) at elevated temperatures (40–75 °C) with an electric current of 11–24 A/dm^3^ applied through the terminals. This process creates an optimal polished finish and a chromium oxide layer. Before this step, stainless steel must be CNC machined according to computer-aided design (CAD) software, and threads are formed using electric discharge machining (EDM), as described in regulations. Even minor deviations during production, such as excess vibration, wobble, inadequate cooling, or spindle torque variation, result in differences in the final product. The numerous steps and machinery involved inevitably lead to product variance.

Measuring very small screws is challenging but feasible using magnification. Metrology-based software ensures proper measurement, yet operator input introduces a tolerance range. In this study, the microscope tolerance was ±0.02 mm. Human accuracy must also be considered, as measurement points are manually determined. The authors standardized measurement points to minimize human variance. They acknowledge that some degree of human error exists; however, this was quantified and found to be consistently positive or above the target measurement. ISO 5835 tolerances are never positive (6); therefore, this operator error has minimal impact on final results. Considering the operator error (+0.03 mm for length and major diameter, +0.01 mm for pitch and minor diameter) and the microscope error (±0.02 mm), recalculated ranges would yield: length +9 screws, major diameter +11, minor diameter +26, pitch no change, angle N/A, polish N/A. Error margins for angle measurement could not be calculated, so those values remain unadjusted. Even accounting for these factors, results remain significant. The majority of screws were outside the tolerance range, indicating low-quality standards, and they did not meet expected measurements.

No set tolerances exist for veterinary implants due to lack of regulation. By selecting a deemed reasonable tolerance, the authors acknowledge a potential study limitation. However, the tolerances chosen were based on human regulations and are not arbitrary. Further research is required to establish permissible tolerances for veterinary implants. The tolerance figures used in this study can serve as preliminary guidelines for future investigations, as this represents the first research into this area of veterinary implants. Manufacturer-level analysis could further clarify whether tolerance deviations cluster by producer, however, incomplete batch-level documentation in routine clinical inventory limited such stratification in the present study. Surface defect assessment was based on a semi-quantitative visual grading system performed by a single observer and was therefore inherently subjective. Although this approach reflects routine quality control practice, future studies incorporating blinded multi-observer scoring or quantitative surface roughness metrics (e.g., Ra) would further strengthen objectivity.

In conclusion, there is no regulation of the veterinary implant industry, and it is evident that there is little oversight or scrutiny of implant quality. This study clearly indicates that the majority of screws measured do not meet established quality standards or expected specifications.

## Data Availability

The original contributions presented in the study are included in the article/Supplementary material, further inquiries can be directed to the corresponding author.
