# Supplementary material for: Tolerance and surface analysis of veterinary bone screws
Source: Front Vet Sci. 2026 Feb 11;13:1723402. doi: 10.3389/fvets.2026.1723402 (PMC12932227; doi:10.3389/fvets.2026.1723402)
Supplement: Supplementary file 1 [file Table_1.docx]

| **Length**  **(mm)** | **Major Diameter D1 (mm)** | **Minor Diameter (mm)** | **Pitch (mm)** | **Combined thread angle (°)** | **Surface grade (1–3)** |
| --- | --- | --- | --- | --- | --- |
| 14.66 | 2.18 | 1.50 | 0.43 | 33.79 | **2** |
| 14.64 | 2.08 | 1.60 | 0.43 | 39.29 | 1 |
| 14.94 | 2.03 | 1.55 | 0.44 | 34.72 | 1 |
| 14.95 | 2.08 | 1.65 | 0.42 | 33.91 | 2 |
| 14.80 | 2.03 | 1.45 | 0.44 | 32.88 | 1 |
| 14.70 | 2.08 | 1.43 | 0.47 | 32.44 | 1 |
| 14.08 | 2.03 | 1.45 | 0.45 | 34.43 | 3 |
| 14.07 | 2.08 | 1.40 | 0.45 | 37.77 | 3 |
| 14.00 | 1.98 | 1.53 | 0.40 | 34.81 | 1 |
| 14.20 | 2.00 | 1.49 | 0.48 | 33.21 | 2 |
| 14.55 | 2.10 | 1.43 | 0.47 | 33.77 | 1 |
| 14.25 | 2.05 | 1.43 | 0.45 | 31.08 | 1 |
| 14.04 | 2.06 | 1.39 | 0.39 | 30.09 | 1 |
| 14.06 | 2.07 | 1.38 | 0.39 | 33.69 | 2 |
| 14.12 | 2.10 | 1.31 | 0.37 | 36.15 | 1 |
| 14.66 | 2.02 | 1.42 | 0.44 | 32.01 | 1 |
| 14.71 | 2.13 | 1.43 | 0.42 | 33.44 | 1 |
| 14.90 | 2.16 | 1.40 | 0.39 | 34.76 | 3 |
| 14.97 | 2.10 | 1.40 | 0.37 | 32.88 | 2 |
| 14.89 | 2.16 | 1.42 | 0.44 | 32.43 | 1 |
| 14.92 | 2.10 | 1.43 | 0.39 | 33.11 | 3 |
| 14.93 | 2.13 | 1.42 | 0.44 | 33.45 | 2 |
| 14.91 | 2.13 | 1.32 | 0.42 | 33.69 | 1 |
| 14.92 | 2.10 | 1.39 | 0.42 | 31.22 | 1 |
| 14.93 | 2.19 | 1.47 | 0.42 | 33.55 | 2 |
| 14.83 | 2.16 | 1.42 | 0.47 | 32.99 | 1 |
| 14.89 | 2.16 | 1.37 | 0.42 | 32.87 | 1 |
| 14.55 | 2.12 | 1.39 | 0.39 | 33.78 | 2 |
| 14.04 | 2.13 | 1.38 | 0.38 | 31.99 | 1 |
| 13.43 | 1.92 | 1.42 | 0.37 | 35.88 | 1 |
| 13.44 | 1.95 | 1.42 | 0.33 | 33.54 | 1 |
| 13.25 | 1.92 | 1.33 | 0.37 | 36.11 | 1 |
| 13.25 | 1.90 | 1.34 | 0.33 | 33.29 | 3 |
| 13.48 | 1.90 | 1.33 | 0.30 | 39.29 | 2 |
| 13.50 | 1.90 | 1.26 | 0.30 | 34.72 | 1 |
| 13.36 | 1.88 | 1.24 | 0.34 | 33.91 | 1 |
| 13.31 | 1.90 | 1.37 | 0.31 | 32.88 | 1 |
| 13.40 | 1.92 | 1.29 | 0.34 | 32.44 | 2 |
| 13.39 | 1.80 | 1.29 | 0.37 | 34.43 | 1 |
| 13.37 | 1.82 | 1.22 | 0.39 | 37.77 | 3 |
| 13.31 | 1.87 | 1.29 | 0.39 | 34.81 | 1 |
| 13.44 | 1.89 | 1.29 | 0.4 | 33.26 | 2 |
| 13.39 | 1.92 | 1.32 | 0.37 | 33.77 | 3 |
| 13.34 | 1.95 | 1.32 | 0.36 | 31.08 | 1 |
| 13.34 | 1.92 | 1.37 | 0.44 | 32.09 | 1 |
| 13.31 | 1.90 | 1.33 | 0.47 | 33.69 | 2 |
| 13.34 | 1.92 | 1.31 | 0.4 | 36.15 | 2 |
| 13.31 | 1.93 | 1.32 | 0.41 | 32.21 | 2 |
| 13.42 | 1.88 | 1.37 | 0.39 | 33.46 | 1 |
| 13.4 | 1.90 | 1.29 | 0.42 | 36.15 | 2 |
| 13.24 | 1.89 | 1.31 | 0.45 | 33.29 | 1 |
| 13.31 | 1.90 | 1.29 | 0.39 | 36.29 | 2 |
| 13.34 | 1.89 | 1.30 | 0.44 | 34.72 | 1 |
| 13.31 | 1.91 | 1.31 | 0.47 | 33.94 | 2 |
| 14.71 | 2.13 | 1.43 | 0.42 | 32.88 | 1 |
| 14.90 | 2.16 | 1.40 | 0.39 | 32.44 | 1 |
| 14.97 | 2.14 | 1.43 | 0.37 | 34.33 | 2 |
| 14.89 | 2.16 | 1.42 | 0.43 | 37.17 | 1 |
| 14.94 | 2.10 | 1.43 | 0.39 | 37.77 | 1 |
| 14.93 | 2.13 | 1.42 | 0.44 | 34.86 | 1 |
| 14.91 | 2.13 | 1.32 | 0.42 | 33.26 | 1 |
| 14.92 | 2.10 | 1.39 | 0.42 | 33.78 | 1 |
| 14.93 | 2.19 | 1.46 | 0.41 | 31.88 | 3 |
| 14.83 | 2.18 | 1.42 | 0.47 | 32.89 | 1 |
| 14.88 | 2.16 | 1.37 | 0.42 | 33.69 | 1 |
| 14.55 | 2.12 | 1.39 | 0.39 | 35.15 | 2 |
| 14.04 | 2.13 | 1.38 | 0.38 | 33.21 | 1 |
| 13.45 | 1.92 | 1.42 | 0.37 | 33.48 | 2 |
| 13.44 | 1.93 | 1.44 | 0.32 | 36.65 | 1 |
| 13.66 | 1.99 | 1.34 | 0.33 | 33.39 | 1 |
| 14.09 | 2.10 | 1.37 | 0.35 | 35.19 | 2 |
| 14.12 | 2.16 | 1.49 | 0.41 | 33.33 | 1 |
| 14.76 | 2.11 | 1.51 | 0.36 | 34.43 | 1 |

**Supplementary Table S2A.** Raw dimensional measurements of 2.0 mm cortical screws (n = 73). Surface grade refers to the semi-quantitative visual grading scale (1 = minor defects, 2 = moderate defects, 3 = major defects).
