# Supplementary material for: Tolerance and surface analysis of veterinary bone screws
Source: Front Vet Sci. 2026 Feb 11;13:1723402. doi: 10.3389/fvets.2026.1723402 (PMC12932227; doi:10.3389/fvets.2026.1723402)
Supplement: Supplementary file 2 [file Table_2.docx]

| Length (Y/N) | Major diameter D1 (Y/N) | Minor diameter (Y/N) | Pitch | Combined thread angle (Y/N) | Surface acceptable |
| --- | --- | --- | --- | --- | --- |
| N | N | N | N | N | **Y** |
| N | N | N | N | N | N |
| N | N | N | N | N | N |
| N | N | N | N | N | Y |
| N | N | N | N | N | N |
| N | N | N | N | N | N |
| N | N | N | N | N | Y |
| N | N | Y | N | Y | Y |
| Y | Y | N | N | N | N |
| N | Y | N | Y | N | Y |
| N | N | N | Y | N | N |
| N | N | N | N | N | N |
| N | N | Y | N | N | N |
| N | N | Y | N | N | Y |
| N | N | N | N | Y | N |
| N | N | N | N | N | N |
| N | N | N | N | N | N |
| N | N | Y | N | N | Y |
| N | N | Y | N | N | Y |
| N | N | N | N | N | N |
| N | N | N | N | N | Y |
| N | N | N | N | N | Y |
| N | N | N | N | N | N |
| N | N | Y | N | N | N |
| N | N | N | N | N | Y |
| N | N | N | Y | N | N |
| N | N | Y | N | N | Y |
| N | N | Y | N | N | N |
| N | N | Y | N | N | N |
| Y | Y | N | N | N | N |
| Y | Y | N | N | N | N |
| N | Y | Y | N | Y | N |
| N | Y | Y | N | N | Y |
| Y | Y | Y | N | N | Y |
| Y | Y | N | N | N | N |
| Y | N | N | N | N | N |
| Y | Y | N | N | N | N |
| Y | N | N | N | N | Y |
| Y | N | N | N | N | N |
| Y | N | N | N | Y | Y |
| Y | N | N | N | N | N |
| Y | N | N | N | N | Y |
| Y | Y | N | N | N | Y |
| Y | Y | N | N | N | N |
| Y | Y | Y | N | N | N |
| Y | Y | Y | Y | N | Y |
| Y | Y | N | N | Y | Y |
| Y | Y | N | N | N | Y |
| Y | N | Y | N | N | N |
| Y | Y | N | N | Y | Y |
| N | N | N | N | N | N |
| Y | Y | N | N | Y | Y |
| Y | N | N | N | N | N |
| Y | Y | N | Y | N | Y |
| N | N | N | N | N | N |
| N | N | Y | N | N | N |
| N | N | N | N | N | Y |
| N | N | N | N | Y | N |
| N | N | N | N | Y | N |
| N | N | N | N | N | N |
| N | N | N | N | N | N |
| N | N | Y | N | N | N |
| N | N | N | N | N | Y |
| N | N | N | Y | N | N |
| N | N | Y | N | N | N |
| N | N | Y | N | N | Y |
| N | N | Y | N | N | N |
| Y | Y | N | N | N | Y |
| Y | Y | N | N | Y | N |
| Y | Y | Y | N | N | N |
| N | N | Y | N | N | Y |

**Supplementary Table S2B.** Tolerance conformity and surface acceptability of 2.0 mm cortical screws (n = 73). Tolerance outcomes are reported as binary pass/fail indicators (Y/N) derived from the original tolerance assessment. Surface acceptability is reported separately (Y/N).
